# *PSORS1C1* Hypomethylation Is Associated with Allopurinol-Induced Severe Cutaneous Adverse Reactions during Disease Onset Period: A Multicenter Retrospective Case-Control Clinical Study in Han Chinese

**DOI:** 10.3389/fphar.2017.00923

**Published:** 2018-01-17

**Authors:** Bao Sun, Lin Cheng, Yan Xiong, Lei Hu, Zhiying Luo, Maosong Zhou, Ji Li, Hongfu Xie, Fazhong He, Xiaoqing Yuan, Xiaoping Chen, Hong-Hao Zhou, Zhaoqian Liu, Xiang Chen, Wei Zhang

**Affiliations:** ^1^Department of Clinical Pharmacology, Xiangya Hospital, Central South University, Changsha, China; ^2^Hunan Key Laboratory of Pharmacogenetics, Institute of Clinical Pharmacology, Central South University, Changsha, China; ^3^State Key Laboratory of Ophthalmology, Zhongshan Ophthalmic Center, Sun Yat-sen University, Guangzhou, China; ^4^Shenzhen Eyeis Visual Science Research Institute, Shenzhen, China; ^5^Department of Pharmacy, Peking University People's Hospital, Beijing, China; ^6^Department of Dermatology, Xiangya Hospital, Central South University, Changsha, China

**Keywords:** allopurinol-SCARs, DNA methylation, HM450, methylated genes, *PSORS1C1*

## Abstract

**Background:** Allopurinol-induced severe cutaneous adverse reactions (SCARs), including drug rash with eosinophilia and systemic symptoms (DRESS), Stevens-Johnson syndrome (SJS) and toxic epidermal necrosis (TEN), are life-threatening autoimmune reactions. Evidence is growing that epigenetic variation, particularly DNA methylation, is associated with autoimmune diseases. However, the potential role of aberrant DNA methylation in allopurinol-SCARs is largely unknown.

**Objective:** To address the knowledge gap between allopurinol-SCARs and DNA methylation, we studied the DNA methylation profiles in peripheral blood cells from allopurinol-SCARs and allopurinol-tolerant subjects.

**Methods:** A genome-scale DNA methylation profiling was conducted using the Illumina Infinium HumanMethylation450 (HM450) platform on 15 patients with allopurinol-SCARs (3 TEN, 2 SJS/TEN overlap and 10 SJS) and 20 age- and gender-matched allopurinol-tolerant controls at disease onset. Pyrosequencing was used to validate the candidate CpG (cytosine-guanine dinucleotide) sites in an independent cohort of 40 allopurinol-SCARs and 48 allopurinol-tolerants.

**Results:** After bioinformatics analysis of methylation data obtained from HM450 BeadChip, we identified 41 differentially methylated CpG loci (*P* < 0.05) annotated to 26 genes showing altered DNA methylation between allopurinol-SCARs and allopurinol-tolerants. Among these genes, significant hypomethylation of *PSORS1C1* (cg24926791) was further validated in a larger sample cohort, showing significant difference between DRESS and controls (*P* = 0.00127), ST (SJS and TEN) and controls (*P* = 3.75 × 10^−13^), and SCARs and controls (*P* = 5.93 × 10^−15^).

**Conclusions:** Our data identified differentially methylated genes between allopurinol-SCARs and allopurinol-tolerant controls and showed that *PSORS1C1* hypomethylation was associated with allopurinol-SCARs (OR = 30.22, 95%CI = 4.73–192.96) during disease onset, suggesting that aberrant DNA methylation may be a mechanism of allopurinol-SCARs.

**Limitations:** Firstly, the data come from whole blood samples known to possess epigenetic heterogeneity, i. e., blood samples comprise a heterogeneous cell population with varying proportions of distinct cell-types with different DNA methylation patterns. Consequently, the interpretation of DNA methylation results should be performed with great caution due to the heterogeneous nature of the sample. Secondly, whether the identified disease-associated changes of epigenome precede disease onset, or result from the disease progression, needs further investigation. Comparing the methylation status before patients develop allopurinol-SCARs and after may help examine methylation levels from disease onset to disease progression.

Allopurinol, an uric acid-decreasing drug widely used for hyperuricemia and its complications, can cause severe cutaneous adverse reactions (SCARs), which includes drug rash with eosinophilia and systemic symptoms (DRESS), Stevens-Johnson syndrome (SJS) and toxic epidermal necrosis (TEN) (Ghislain and Roujeau, 2002). In general, allopurinol-induced hypersensitivity manifests as a mild skin rash in about 2% of users, however, approximately 0.4% will develop into more serious cutaneous adverse reactions, with a high mortality from 9 to 32% (Singer and Wallace, 1986; Arellano and Sacristan, 1993; Halevy et al., 2008; Ramasamy et al., 2013).

Allopurinol-SCARs were considered to be the result of immune-mediated hypersensitivity (Arellano and Sacristan, 1993; Braden et al., 1994; Lin et al., 2015). Genetic factors predisposing to allopurinol-SCARs have been identified particularly in *Human Leukocyte Antigen B* gene (*HLA*-*B*, Gene ID: 3106), allele *HLA*-B^*^5801. In Asian populations, allopurinol-SCARs has been shown to occur almost exclusively in HLA-B^*^5801 positive individuals in Han Chinese of Taiwan, Hong Kong, and mainland China (Hung et al., 2005; Cao et al., 2012; Chiu et al., 2012; Cheng et al., 2015), and Thai descent (Tassaneeyakul et al., 2009). The prior-to-prescription screening of HLA-B^*^5801 was considered to markedly reduce the incidence rate of allopurinol-SCARs in Taiwan (Ko et al., 2015). However, this strategy might not be cost-effective in non-Asians or Japanese. The majority of these people with this allele do not develop allopurinol-SCAR and it is not known why this is the case. The mechanisms underlying allopurinol-SCARs have not been fully elucidated.

Recent studies shed light on epigenetic modification across autoimmune diseases. Systemic Lupus Erythematosus (SLE) was reported to be associated with global T cell hypomethylation (Balada et al., 2007; Zhou and Lu, 2008) and global DNA hypomethylation (Zhu et al., 2011). Aberrant DNA methylation had been implicated in drug-induced lupus (Richardson, 2003; Zhou and Lu, 2008) and autoimmune Addison's disease (Bjanesoy et al., 2014). Hypomethylation in the promoter region of some genes, such as *Integrin Subunit Alpha L* (*ITGAL*, Gene ID: 3683) (Yung et al., 1996; Lu et al., 2002), *Perforin 1* (*PRF1*, Gene ID: 5551) (Lu et al., 2003; Kaplan et al., 2004), and members of *Tumor Necrosis Factor Receptor Superfamily* (*TNFSF5* and *TNFSF7*, Gene ID: 959 and 970, respectively) (Oelke et al., 2004; Lu et al., 2005, 2007), were reported in lupus T cells. The interplay of genetic and environmental factors might be important for the occurrence and development of autoimmune diseases (Ellis et al., 2010). Therefore, interrogation of the epigenome might uncover molecular targets for the diagnosis and prevention of allopurinol-SCARs, and thus deserves further investigation.

Given the increasing evidence for epigenetic disruption in autoimmune diseases, we hypothesized that DNA methylation may be one of the contributing factors that promote allopurinol-SCARs. Therefore, we investigated the DNA methylation status of 484,660 CpG dinucleotides in 15 patients with allopurinol-SCARs and 20 age- and gender-matched allopurinol-tolerant controls using the Illumina Infinium HumanMethylation450 (HM450) platform. Unraveling epigenetic factors in allopurinol-SCARs will not only benefit our understandings of genetic and epigenetic interaction in drug-hypersensitivity, but may also lead to new therapies or discovery of epigenetic biomarkers for the disease.

## Methods

### Study population

The study was registered in the Chinese Clinical Trial Registry with ID# ChiCTR-RCC-12002927 (http://www.chictr.org.cn/showprojen.aspx?proj=6628) and was approved by the Independent Ethics Committee of Institute of Clinical Pharmacology, Central South University (CTXY-110011-2). Patients who had taken allopurinol and were diagnosed with SJS/TEN (SJS, SJS/TEN overlap and TEN) or DRESS by at least two dermatologists according to the consensus diagnostic criteria (Bastuji-Garin et al., 1993; Roujeau, 2005; Kardaun et al., 2007; Cacoub et al., 2011) were enrolled. Briefly, SCARs occurred within 3 months of allopurinol use, with its diminishment or relieved symptoms upon withdrawal. The criteria of DRESS are as follows: rash in combination with fever, lymphadenopathy and hematological abnormalities (e.g., eosinophilia, atypical lymphocytes, leukocytosis, and thrombocytopenia) along with involvement of internal organ (e.g., hepatitis and nephritis). Patients with SJS were defined as skin detachment less than 10% of the total body surface area (BSA), the ones with SJS/TEN overlap were defined as skin detachment between 10 and 30% of BSA, and those with TEN were defined as skin detachment more than 30% of BSA. Details of these cohorts had been described previously (Cheng et al., 2015). Allopurinol-tolerant individuals were defined as patients using allopurinol for at least 3 months without any evidence of cutaneous adverse reactions. Patients with a medical history of bone marrow transplantation, chemotherapy or cancer were excluded. All enrolled participants were of Han Chinese descent and provided written informed consent.

### Study design

The study was designed as a multicenter retrospective case-control clinical study. Patients were recruited from 19 centers across China when the disease occurred. A total of 15 patients with allopurinol-SCARs (3 TEN, 2 SJS/TEN overlap and 10 SJS) and 20 allopurinol-tolerant subjects matched for age and gender were recruited to the study for DNA methylation profiling. Validation of candidate sites was performed in another sample cohort of 40 allopurinol-SCARs and 48 allopurinol-tolerants using pyrosequencing. The blood samples were taken on the first day when allopurinol-SCARs patients were admitted to the hospitals where the treatment was initiated. The rash happened a few days to around 3 weeks before reporting to the hospitals. The samples of allopurinol-tolerant patients were collected during their routine visit to the rheumatology clinics. A flowchart showing the design of the study is presented in Supplementary Figure 1.

### DNA extraction and genome-scale methylation beadchip assay

Genomic DNA was extracted from 2 ml venous blood of each participant followed by centrifugation at 2,500 rpm for 15 min using Wizard® Genomic DNA Purification Kit (Promega, Madison, Wisconsin, USA). The extracted DNA underwent bisulfite conversion by the EZ DNA Methylation™ Kit (Zymo Research) according to the manufacturer's instructions. Converted DNA was then applied to the Illumina Infinium HM450 BeadChip arrays (Illumina, San Diego, CA) and hybridized on BeadChips. Each chip was scanned by a HiScan 2000 (Illumina). The array measured methylation at >480,000 CpG sites in 99% of RefSeq genes. The array incorporated 484,660 probes in total, including the quality control probes.

### Genome-scale methylation data analysis

The raw data of 484,660 probe signal intensities were obtained through the Genome Studio software. After comparing with the different probe florescence and probe types, the raw data were filtered, normalized and corrected. Then the probes were subjected to a second round of filtering by excluding probes targeting the X and Y chromosomes, probes overlapping with single nucleotide polymorphisms (SNPs), probes containing repeat sequences of ≥10 bp. Finally, 360,012 probes were left for differentially expressed methylation comparison through empirical Bayes moderated *t*-test analysis in the package of limma. The *P*-value mentioned herein is the adjusted *P*-value after multiple testing adjustment by false discovery rate (FDR)-controlling procedure. Probes with *P* < 0.05 were considered statistically different between the tested groups. All genes measured by the HM450 were used as the reference set in this analysis.

The methylation level was computed as β-value according to the mean methylated (M) and unmethylated (U) signal intensities for each probe for each sample using the formula (β = M/(M+U+100). To evaluate the exactitude of the obtained β value and remove noise, probes with detection *P*-values of < 0.05 were deemed significantly different from background. We also included an additional filter requiring the magnitude of mean β-value difference ≥ 0.2 between groups for all group comparisons. Data analysis was performed by the statistical programming language R (version 3.1.0) (www.r-project.org) and packages from the Bioconductor project (Gentleman et al., 2004). Two-dimensional hierarchical clustering, Venn diagram and volcano plot of the differential DNA methylation data were performed by the default algorithms in the R software. Given that statistically significant sites might limit biological meaning for small methylation differences, we analyzed the data in the context of *P* < 0.05. Differentially methylated genes underwent Gene Ontology (GO) terms and Kyoto Encyclopedia of Genes and Genomes (KEGG) pathway using the Gene Annotation Tool to Help Explain Relationships (GATHER) webtool (http://changlab.uth.tmc.edu/gather/; Chang and Nevins, 2006). Analysis methods of the study are presented in Supplementary Figure 1.

### DNA methylation validation by bisulfite pyrosequencing

Array data were validated by bisulfite pyrosequencing, which was performed by a PyroMark Q24 ID pyrosequencing instrument according to the manufacturer's instructions (Qiagen). To increase template quantity for pyrosequencing assays, 200 ng of genomic DNA was treated with EZ DNA Methylation™ Kit. Thereafter, amplicons containing CpGs of interest were prepared by PCR using the forward, reverse and sequencing primers, which were designed by PyroMark Assay Design software (Qiagen), specific for bisulfite-converted DNA (assay primers are provided in Supplementary Table 1). The methylation percentage for each CpG within the target sequence was analyzed using the PyroMark Q24 software (v 2.0.6., build 20; Qiagen).

### Statistical analysis

Demographic and baseline characteristics were analyzed using the SPSS16.0 software (SPSS Inc., Chicago, IL). All the numerical variables in this article were presented as mean ± standard deviation (SD). Independent-samples student's *t*-test was used on numerical variables and Chi-square test was used on categorical variables. Two-sided test was applied on all available data. A *P* < 0.05 was considered statistically significant.

## Results

### Allopurinol-SCARs are differentially methylated to allopurinol-tolerant controls

**Figure 1:**
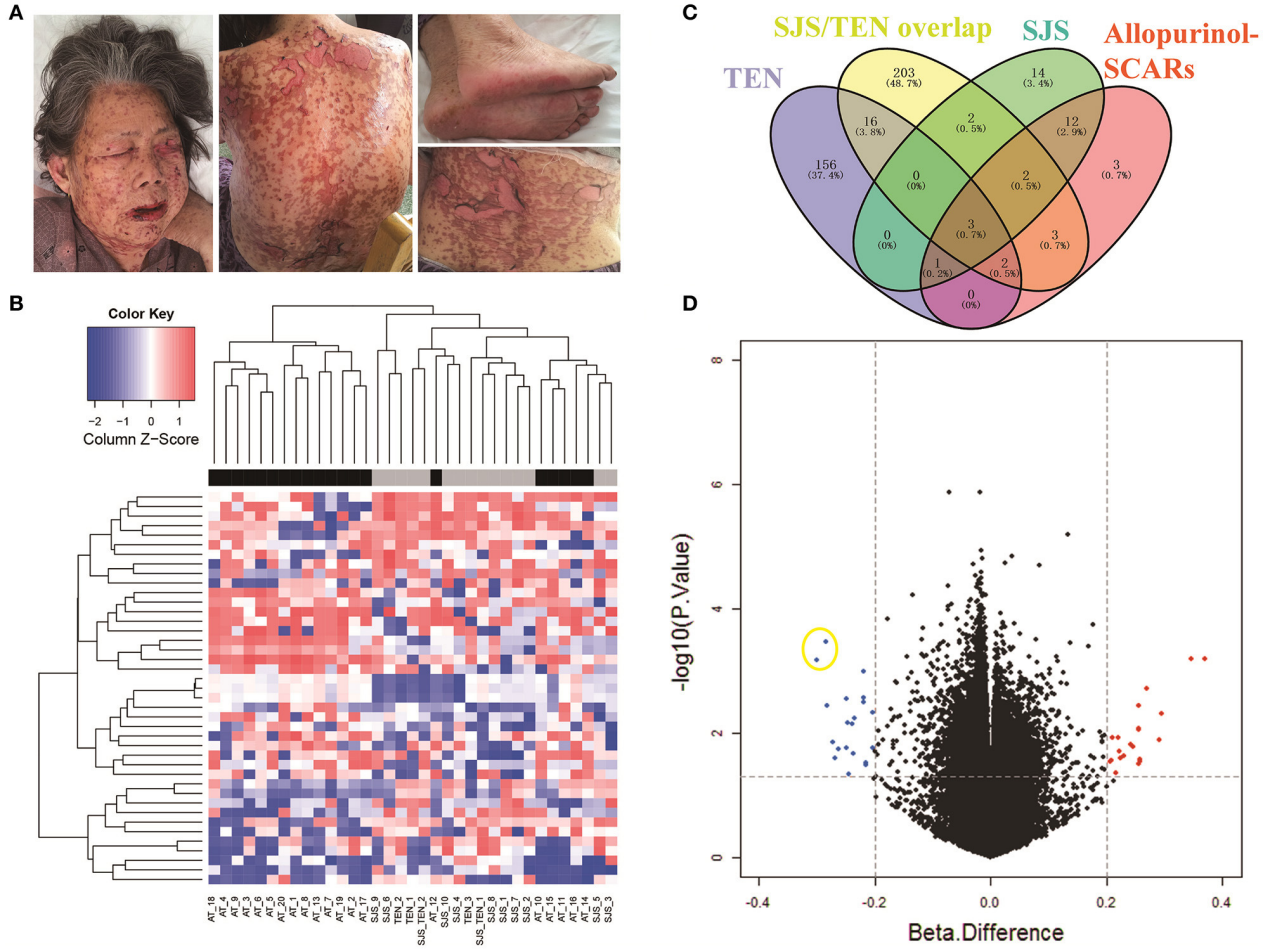
Identification of DNA methylation difference between allopurinol-SCARs and allopurinol-tolerant controls. **(A)** Dermatological manifestations of one featured SJS/TEN overlap patient. The patient has given written consent for the publication of her images. As showed, 10–30% of the total body surface area was involved. Mucocutaneous sloughing was observed in the mouth, eyes and genital area. Epidermal detachment in the face, neck, back and extremities was observed. **(B)** Two-dimensional hierarchical clustering was performed using the statistical variable DNA methylation probes across all samples (*n* = 35). Probes are in rows; samples are in columns. **(C)** Venn diagram showed the overlaps of methylated genes in different phenotypes of allopurinol-SCARs. **(D)** Volcano plot of the differential DNA methylation analysis. X-axis denotes β-value differences; Y-axis denotes *P*-values for each probe (-log_10_ scale). Vertical dotted lines denote 20% change in β-values; horizontal dotted line denotes the significance cutoff. Two hypomethylated dots in yellow circle are the probes of cg08412936 (up) and cg24926791 (down) in *PSORS1C1* promoter area.

**Figure 2:**
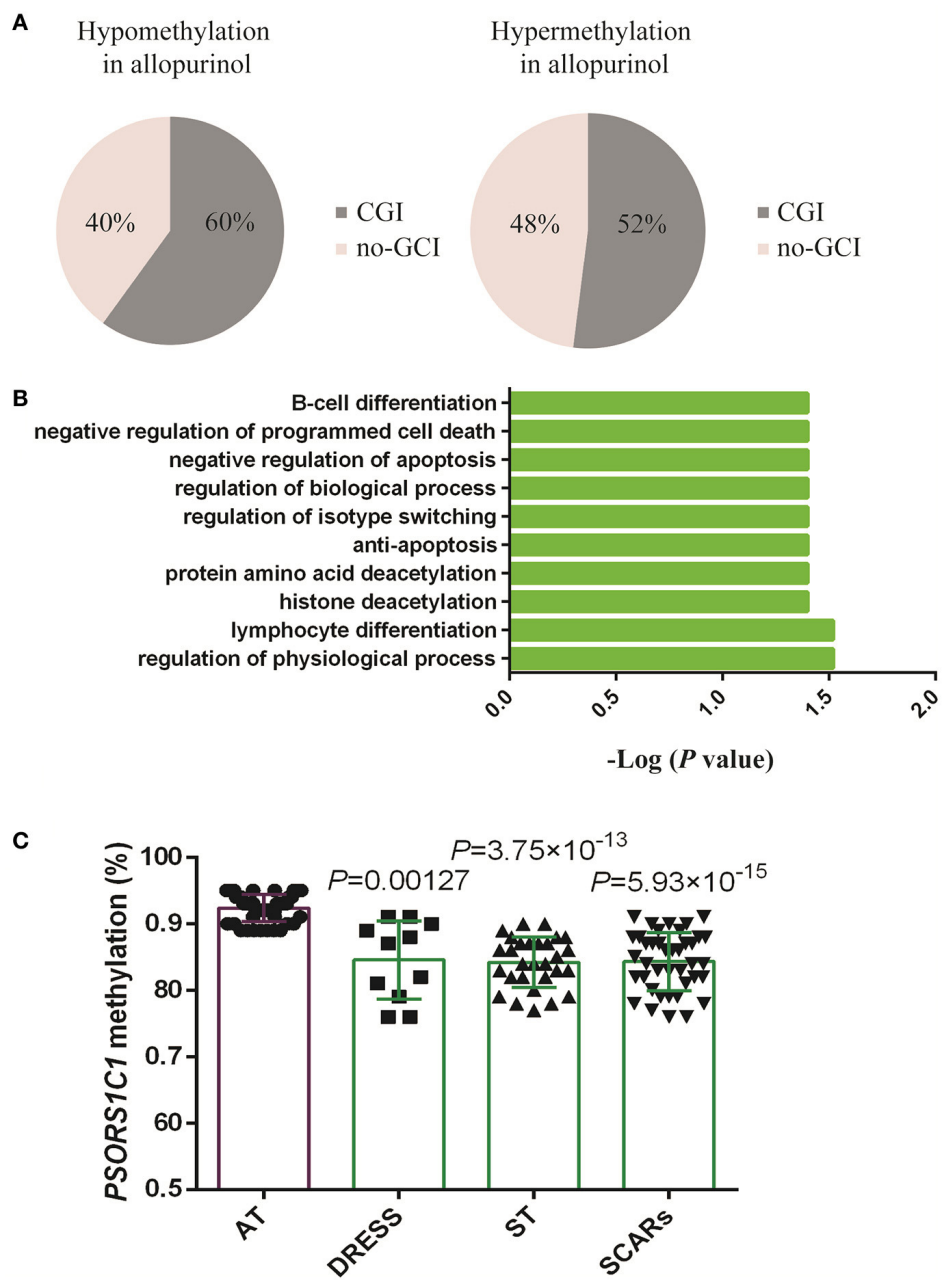
Analysis and validation of differentially methylated gene probes. **(A)** Proportions of gene probes associated with CpG islands (CGI) and no-CGI. **(B)** Gene Ontology (GO) biological processes annotation of differentially methylated genes using the GATHER Functional Annotation tool. **(C)** Validation of differentially methylated array site in gene *PSORS1C1* in the replication cohort. Values were presented as mean ± SD. AT, allopurinol-tolerants; ST included SJS, SJS/TEN overlap and TEN; SCARs included DRESS, SJS, SJS/TEN overlap, and TEN. DRESS vs. AT *P* = 0.00127; ST vs. AT *P* = 3.75 × 10^−13^; SCARs vs. AT *P* = 5.93 × 10^−15^. Student's *t*-test analysis was applied for the comparison.

A featured SJS/TEN overlap patient is shown in Figure 1A. The demographic characteristics of 15 allopurinol-SCARs and 20 allopurinol-tolerant controls analyzed for genome-scale DNA methylation profiles are shown in Table 1. Hierarchical clustering of the statistically different probes that varied most across the allopurinol-SCARs and allopurinol-tolerants was performed and illustrated in Figure 1B. Overall, the DNA methylation profiles of allopurinol-SCARs and allopurinol-tolerants resulted in separate clusters, indicating a substantial difference in DNA methylation profiles between allopurinol-SCARs and allopurinol-tolerants. By using a locus-by-locus differential DNA methylation analysis using a cut-off of *P* < 0.05 and a minimum median β-value difference of 20%, we identified 20 probes (11 genes) with significant hypomethylation and 21 probes (15 genes) with significant hypermethylation in allopurinol-SCARs (Figure 1D; Table 2). To investigate whether the differentially methylated probes were corresponding to genes associated with CpG islands, we found that 60% of hypomethylated probes and 52% of hypermethylated probes were related to CpG islands in allopurinol-SCARs (Figure 2A).

**Table 1:** Demographic characteristics of allopurinol-SCARs and allopurinol-tolerants analyzed for genome-scale DNA methylation profiles.

**Characteristics**	**TEN (*n* = 3)**	**SJS/TEN overlap (*n* = 2)**	**SJS (*n* = 10)**	**SCARs (*n* = 15)** ^**∫**^	**Tolerant controls (*n* = 20)**^**¢**^	**SCARs vs. tolerant controls *P*-value**
**AGE (YEARS)**						
Mean ± SD; range	63.33 ± 14.01; 49–77	65.50 ± 2.12; 62–67	61.33 ± 10.87; 35–74	59.12 ± 1.62; 35–77	58.95 ± 14.59; 37–80	0.599
**GENDER**						
Male, n (%)	2 (66.67)	2 (100)	9 (90)	13 (86.67)	19 (95)	0.565
Female, n (%)	1 (33.33)	0 (0)	1 (10)	2 (13.33)	1 (5)	
ALLOPURINOL EXPOSURE DOSE (mg/Day)^*^						
Mean ± SD; range	100	100	175 ± 95.74; 100–300	150 ± 83.67; 100–300	175 ± 96.53; 100–600	0.035
ALLOPURINOL EXPOSURE TIME (Day)^**^						
Mean ± SD; range	34 ± 23.80; 15–60	9	19.14 ± 11.02; 5–60	22.27 ± 15.72; 5–60	1,629.50 ± 2,803.13; 180–7,300	0.019
**COMORBIDITY (%)**						
Fever, n (%)	2 (66.67)	1 (50)	3 (30)	6 (40)	0 (0)	
Hypertension, n (%)	0 (0)	0 (0)	2 (20)	2 (13.33)	5 (25)	
Hyperglycemia, n (%)	1 (33.33)	1 (50)	3 (30)	5 (33.33)	6 (30)	
RFI, n (%)	1 (33.33)	1 (50)	2 (20)	5 (33.33)	3 (15)	
Miscellany, n (%)	3 (100)	1 (50)	8 (80)	12 (80)	5 (25)	

“*” * Information not available for two subjects*,

“**” * Information not available for one subject*.

“∫” 15 HLA-B^*^5801 positive individuals, carrying one copy,

“¢” 17 HLA-B^*^5801 negative and 3 HLA-B^*^5801 positive individuals (carrying one copy of the allele). RFI: renal function insufficiency.

**Table 2:** Genes significantly methylated in allopurinol-SCARs when compared with allopurinol-tolerant controls.

**Probes**	**Gene name**	**Gene ID**	***P*-value**	**β-value difference**	**Gene region**
cg24926791	*PSORS1C1*	170679	0.00068	−0.3	TSS1500
cg08412936	*PSORS1C1*	170679	0.00034	−0.28	5'UTR
cg19405842	PRKCZ	5590	0.0038	−0.28	5'UTR; Body
cg18997918	SFRS8	6433	0.014	−0.27	Body
cg14408831	–	–	0.025	−0.27	
cg02507579	*OR5H15*	403274	0.018	−0.26	1st Exon
cg12562822	*SLC6A19*	340024	0.018	−0.25	Body
cg12214399	–	–	0.0028	−0.25	
cg17678867	*SYNE1*	23345	0.0071	−0.25	Body
cg11857805	–	–	0.047	−0.24	
cg11791078	*RANBP3L*	202151	0.0073	−0.24	Body
cg27114706	–	–	0.021	−0.24	
cg19245335	*PRDM15*	63977	0.006	−0.23	Body
cg21548813	*DUSP22*	56940	0.0027	−0.22	TSS1500
cg15383120	*DUSP22*	56940	0.0032	−0.22	TSS200
cg20073472	*PSORS1C3*	100130889	0.001	−0.22	TSS1500
cg21645759	–	–	0.031	−0.22	
cg25203245	*FLJ32810*	143872	0.033	−0.21	Body
cg03395511	*DUSP22*	56940	0.0047	−0.2	TSS200
cg17250082	–	–	0.018	−0.2	
cg23878260	*NCOR2*	9612	0.029	0.21	Body
cg26296371	*FARS2*	10667	0.028	0.21	Body
cg26881691	–	–	0.012	0.21	
cg12515659	*FAM134B*	54463	0.045	0.22	Body
cg10568066	*RNF39*	80352	0.012	0.22	Body
cg00501169	*NBPF4*	148545	0.02	0.22	Body
cg13962846	–	–	0.026	0.22	
cg22007216	*DCHS2*	54798	0.023	0.23	Body; 1st Exon
cg05455372	*LOC168474*	168474	0.016	0.24	TSS200
cg05593887	*MAGI2*	9863	0.017	0.25	Body
cg11074353	–	–	0.0087	0.25	
cg12307373	*DYNC2H1*	79659	0.009	0.25	Body
cg10930308	*RNF39*	80352	0.0037	0.26	Body
cg16060930	*PTGFRN*	5738	0.032	0.26	Body
cg05971102	–	–	0.03	0.26	
cg26076233	*DYNLL1*	8655	0.026	0.26	5'UTR
cg00033213	*TOP1MT*	116447	0.002	0.27	Body
cg22138998	*SFRS9*	8683	0.013	0.29	Body
cg05412957	*SCGN*	10590	0.0049	0.3	3'UTR
cg26035071	–	–	0.00065	0.35	
cg26077133	*MSRA*	4482	0.00064	0.37	Body

*UTR, Untranslated Region; TSS, Transcriptional start site; TSS1500, a site located 1,500 bp upstream of the start codon of the gene; Body: gene region. “–”, no gene is corresponding to the probe. The gray highlighted probes are selected for pyrosequencing validation in an independent cohort of samples*.

We next conducted a locus-by-locus differential DNA methylation analysis in each phenotype of allopurinol-SCARs under the condition of *P* < 0.05 and a minimum median β-value difference of 20%, and found 274 methylated probes (178 genes) in TEN, 339 methylated probes (231 genes) in SJS/TEN overlap, and 54 methylated probes (34 genes) in SJS, respectively (Figure 1C). We found statistically significant overlaps (all *P* < 0.05) of methylated genes in different phenotypes of allopurinol-SCARs, among which *psoriasis susceptibility 1 candidate 1* (*PSORS1C1*, Gene ID: 170679), *protein kinase C zeta* (*PRKCZ*, Gene ID: 5590) and *dual specificity phosphatase 22* (*DUSP22*, Gene ID: 56940) were hypomethylated in each phenotype of allopurinol-SCARs.

In order to understand the features of these epigenetic regulated genes, a GATHER functional annotation analysis was performed. It indicated that the differentially methylated set of genes was significantly enriched in GO biological processes including regulation of physiological process, lymphocyte differentiation, histone deacetylation, protein amino acid deacetylation, anti-apoptosis, regulation of isotype switching, regulation of biological process, and negative regulation of apoptosis (*P* < 0.05, results obtained from choosing “inter from network”). The top 10 GO annotations are shown in Figure 2B. In addition, GATHER found two KEGG pathways JAK-STAT signaling pathway and Notch signaling pathway were significantly associated with allopurinol-SCARs (*P* < 0.05), with 6 or 3 genes in each pathway.

### *PSORS1C1* hypomethylation is validated in an independent cohort of allopurinol-SCARs

To ensure that our findings were not dependent on the specific population analyzed, we performed gene-specific DNA methylation analysis on an independent cohort of 40 allopurinol-SCARs and 48 allopurinol-tolerant controls (Supplementary Table 2) through bisulfite pyrosequencing. Two candidate CpG sites located at *PSORS1C1* and *DUSP22* were selected for validation due to their location upstream of the transcriptional start site (TSS) of each gene, which are more likely to interfere with the function of promoters. Assay primers were provided in Supplementary Table 1.

The *PSORS1C1* cg24926791 CpG site not only had the large HM450 case-control β-value difference in each phenotype of allopurinol-SCARs, but had also been associated with the development of autoimmune diseases (Sun et al., 2013) as reported previously. We quantitatively measured methylation at three CpG dinucleotides of *PSORS1C1*, including the HM450 differentially methylated probe site (array site), CpG 1 and CpG2. CpG 1 and CpG2 were unintentionally found locus after the array site (Supplementary Figures 2A,B). The methylation levels were observed in pyrosequencing CpG sites (Table 3). As presented in Figure 2C, DRESS that was not included in the DNA methylation profiling showed decreased methylation in the replication samples compared to the allopurinol-tolerant controls (*P* = 0.00127 vs. *P* = 3.75 × 10^−13^, *P* = 5.93 × 10^−15^), which imply that DRESS is less in association with *PSORS1C1* hypomethylation. The results suggest that DRESS and SJS/TEN are parts of the same spectrum of diseases but with less severity. In parallel to the testing cohort, we combined the data of SJS, SJS/TEN overlap and TEN, and found the same tendency of methylation changes compared to the allopurinol-tolerant controls (ST vs. controls, *P* = 3.75 × 10^−13^). As expected, we observed that all the cases manifested hypomethylation in the validation samples compared to the controls (SCARs vs. controls, *P* = 5.93 × 10^−15^). Patients with *PSORS1C1* methylation level lower than 90% had 30.22-fold (95%CI = 4.73–192.96, *P* < 0.001) higher risk to develop allopurinol-SCARs than allopurinol-tolerants (Table 4).

**Table 3:** Pyrosequencing validation of methylation levels for *PSORS1C1* cg24926791 CpG site in an independent 40 allopurinol-SCARs and 48 allopurinol-tolerants cohort.

**Allopurinol-SCARs**	**Array site**	**CpG1**	**CpG2**	**Allopurinol-tolerants**	**Array site**	**CpG1**	**CpG2**
DRESS	0.87	0.96	0.93	AT	0.95	1	0.97
DRESS	0.91	1	0.94	AT	0.91	0.95	0.93
DRESS	0.91	1	0.89	AT	0.93	0.96	0.93
DRESS	0.76	1	0.84	AT	0.93	0.92	0.95
DRESS	0.88	1	0.89	AT	0.95	1	0.96
DRESS	0.81	1	0.85	AT	0.92	0.95	0.89
DRESS	0.89	0.98	0.94	AT	0.92	−	−
DRESS	0.9	1	0.93	AT	0.95	1	0.99
DRESS	0.79	0.86	0.79	AT	0.93	1	0.97
DRESS	0.82	0.92	0.86	AT	0.93	0.92	0.92
DRESS	0.76	0.89	0.84	AT	0.95	1	0.97
SJS	0.87	1	0.88	AT	0.93	1	0.96
SJS	0.9	0.95	0.89	AT	0.94	0.97	0.98
SJS	0.87	0.94	0.89	AT	0.95	1	0.95
SJS	0.77	0.9	0.88	AT	0.92	−	−
SJS	0.87	0.93	0.9	AT	0.94	0.9	0.93
SJS	0.82	0.93	0.85	AT	0.92	1	0.92
SJS	0.83	0.93	0.82	AT	0.93	1	0.96
SJS	0.85	1	0.91	AT	0.94	1	0.97
SJS	0.87	0.93	0.9	AT	0.92	1	0.94
SJS	0.84	0.91	0.8	AT	0.94	1	0.98
SJS	0.78	0.89	0.8	AT	0.95	1	0.97
SJS	0.82	0.9	0.86	AT	0.92	0.89	0.89
SJS	0.86	0.91	0.87	AT	0.95	1	1
SJS	0.78	0.9	0.8	AT	0.9	0.92	0.91
SJS	0.86	0.9	0.86	AT	0.92	−	−
SJS	0.88	0.9	0.89	AT	0.93	1	0.95
SJS	0.83	0.92	0.87	AT	0.92	0.95	0.86
SJS	0.82	0.9	0.82	AT	0.89	1	0.87
SJS	0.8	0.89	0.8	AT	0.89	1	0.89
SJS	0.83	0.2	0.84	AT	0.95	0.95	0.96
SJS	0.79	0.9	0.8	AT	0.94	1	0.94
SJS/TEN overlap	0.86	0.99	0.91	AT	0.93	0.95	0.9
SJS/TEN overlap	0.9	0.94	0.9	AT	0.95	0.98	0.92
SJS/TEN overlap	0.84	0.93	0.86	AT	0.92	0.93	0.94
SJS/TEN overlap	0.84	0.92	0.87	AT	0.95	0.99	0.93
TEN	0.89	1	0.91	AT	0.93	1	0.92
TEN	0.88	0.96	0.95	AT	0.91	0.99	0.94
TEN	0.79	0.93	0.9	AT	0.89	0.92	0.92
TEN	0.88	0.92	0.88	AT	0.9	0.94	0.94
				AT	0.9	0.91	0.91
				AT	0.93	0.93	0.94
				AT	0.91	0.92	0.93
				AT	0.89	−	−
				AT	0.89	0.91	0.95
				AT	0.89	0.91	0.9
				AT	0.89	0.91	0.9
				AT	0.9	0.92	0.92

*AT, allopurinol-tolerants; “–”, missing data (did not pass quality control); array site, the HM450 differentially methylated PSORS1C1 cg24926791 CpG site. When using pyrosequencing to validate the array site, there were two CpG loci spontaneously found immediately after the CpG array site. These two sites were named as CpG1 and CpG2*.

**Table 4:** Association between variables and allopurinol-SCARs risk.

**Predictor variables**	**Unadjusted OR (95% CI)**	***P-*value**	**Adjusted OR (95% CI)**	***P-*value**
Age (>58 vs. ≤ 58)	1.12 (0.485–2.609)	0.78	0.47 (0.08–2.54)	0.38
Gender (male vs. female)	0.588 (0.197–1.753)	0.34	1.295 (0.14–11.61)	0.82
HLA-B^*^5801 (+ vs. –)	63 (16.48–240.89)	< 0.001	38.01 (7.00–206.31)	< 0.001
*PSORS1C1* methylation levels (≤ 0.9 vs. >0.9)	63.91 (13.25–308.16)	< 0.001	30.22 (4.73–192.96)	< 0.001

OR, odds ratio; CI, confidence interval; +, HLA-B^*^5801 positive individuals; –, HLA-B^*^5801 negative individuals. HLA-B^*^5801 (+) allopurinol-SCARs patients have one copy of HLA-B^*^5801.

We also quantitatively measured methylation at *DUSP22*, however, we failed to find the parallel methylation changes in the independent validation cohort (Supplementary Table 3, Supplementary Figure 2C). The methylation level of cg03395511 CpG site in *DUSP22* showed no significant difference between cases and controls (*P* > 0.05).

## Discussion

Using genome-level interrogation of DNA methylation, we present the first genome-scale analysis of DNA methylation profile in allopurinol-SCARs. We identified 26 differentially methylated genes in allopurinol-SCARs, which may be helpful in finding useful epigenetic biomarkers for drug-induced adverse reactions. In our study, few loci were captured to have statistically significant associations with allopurinol-SCARs by using an adjusted *P* < 0.05 for differential methylation. Since DNA methylation studies in complex diseases did not obtain the same broad range of variation as cancer studies (Selamat et al., 2012), our approach focused on associations under the condition of *P* < 0.05, thus revealing sites more likely to be biologically meaningful.

Of the differentially methylated loci, *PSORS1C1*, a susceptibility gene for psoriasis, stood out as an interesting case. Previous researches revealed a significant increase in expression of *PSORS1C1* in rheumatoid arthritis (RA) synovial tissues, indicating that *PSORS1C1* might play an important role in the development of RA (Sun et al., 2013). In Japan, SNP (rs9263726) in *PSORS1C1* gene is in absolute linkage disequilibrium with HLA-B^*^5801, which is strongly associated with allopurinol-SJS/TEN (Tohkin et al., 2013). It is not known if this SNP in *PSORS1C1* is in linkage disequilibrium with HLA-B^*^5801 in Han Chinese. In our study, *PSORS1C1* hypomethylation is closely associated with allopurinol-SCARs, possibly because hypomethylation opens up *PSORS1C1* and thus provides conjunction binding site for HLA-B^*^5801. The epigenetic modification of *DUSP22* in drug-hypersensitivity is interesting for further investigation, although not validated by the bisulfite pyrosequencing analysis. Previous study showed that *DUSP22* was overexpressed in activated immune cells. Moreover, mice with *DUSP22* suppression presented a significant reduction in inflammatory responses (Hamamura et al., 2015). The reason we fail to validate *DUSP22* cg03395511 in a larger cohort may due to the heterogeneity of samples. The significant BeadChip arrays data cannot always be validated in a large sample set. In addition, *PRKCZ*, though not included in our bisulfite pyrosequencing validation analysis, was reported before to be associated with the metabolism of serum vitamin D and relapse in multiple sclerosis (Lin et al., 2014). This gene can be further assessed for its potential function in allopurinol-SCARs by future studies.

Moreover, the gene ontology analysis of differentially methylated genes suggested autoimmune disease-relevant biological processes including lymphocyte differentiation, anti-apoptosis, regulation of isotype switching, and negative regulation of apoptosis were significantly associated with allopurinol-SCARs. These differentially methylated genes were enriched in the JAK-STAT and Notch signaling pathways after KEGG analysis. The two pathways are involved in immunity, proliferation, differentiation, and apoptosis. Disruption or deregulation of these two pathways can result in immune response dysfunction. The relevance of those two pathways may be further investigated in the future.

In terms of mechanism, whether drug-hypersensitivity with different severity harbors the same pathogenesis remains controversial. Previous studies suggested there was potential difference between the mechanisms of carbamazepine-induced hypersensitivity syndrome (CBZ-HSS) and CBZ-SJS (Amstutz et al., 2013). In our study, we identified some overlapped differentially methylated genes among different phenotypes of allopurinol-SCARs (Figure 1C), indicating that allopurinol-SCARs belonged to a complex disease with high heterogeneity among patients. It is noteworthy that, when we included DRESS samples into the replication cohort, we observed a significant difference from controls, suggesting that DRESS, SJS and TEN might be within the same spectrum of diseases but with less severity. This is consistent with the model of a spectrum of disease severity.

An important issue that needs to be addressed in our study is whether the identified disease-related epigenome changes precede disease onset, or result from the disease progression. The blood samples of allopurinol-SCARs patients were taken after developing adverse reactions. The aim of our study was, in the first instance, to retrospectively review their epigenomic traits that may make patients susceptible to develop drug hypersensitivity. However, we can only know who the patients are after onset of the disease and when they are admitted to the hospital. There are a few days of delay between onset of the adverse reactions and admission to the hospital. So, we presume that the differential methylation of *PSORS1C1* is a pre-existing trait that increases susceptibility to allopurinol-SCARs. If this is the case, *PSORS1C1* hypomethylation is the risk factor that can be utilized to predict the susceptible patients before they are admitted to the hospital. But compared with *PSORS1C1* hypomethylation, it is obvious that HLA-B^*^5801 is a much more reliable susceptibility marker for allopurinol-SCARs, as it has a much higher positive predictive value, negative predictive value and significance. Moreover, as a genetic factor, allele HLA-B^*^5801 is easier to detect via genotyping of DNA isolated from whole blood, with no need for biopsy of the susceptible tissue. It is however possible that the observed epigenomic alterations may be the result of disease progression, which dynamically change along with the severity of the disease. Thus, it could be a predictor performed in the affected tissue while therapy for the disease is still in progress to know the severity and prognosis of the disease. This test could be done during the onset of the disease to monitor possible adverse reactions and the treatment effect. In any case, all the patients are selected during disease onset period in our study, demonstrating that allopurinol-SCARs are associated with aberrantly methylated genes. Aberrant DNA methylation indicates that such changes may contribute to the disease and may help to understand possible underlying mechanisms. Further studies could measure the methylation levels before, during and after the disease to examine changes during disease onset and progression.

Our study has limitations. We only investigated peripheral blood for ascertaining DNA methylation, since it is difficult to obtain skin tissue samples from patients with allopurinol-SCARs. Blood may not be an ideal surrogate tissue, despite recent researches support the utilization of peripheral blood lymphocyte DNA to detect epigenetic biomarkers of organ-specific diseases (Anglim et al., 2008; Sinnaeve et al., 2009). The study would be of more significance if the experiments were performed using genomic DNA isolated from specific immune cell populations. In the future, it will be rational to compare epigenetic alterations among different tissue samples from allopurinol-SCAR individuals.

## Conclusions

In conclusion, our study demonstrated a genome-scale difference in peripheral blood DNA methylation profiles between allopurinol-SCARs and allopurinol-tolerant controls when the disease occurred. Our results demonstrated that hypomethylation of *PSORS1C1* was significantly associated with the development of allopurinol-SCARs, suggesting that aberrant DNA methylation may be a mechanism of allopurinol-SCARs.

## Author contributions

BS and LC performed lab experiments, analyzed the data and wrote the paper; JL and HX collected clinical patient's information; YX, LH, ZL, MZ, FH and XY collected DNA samples and made records; XiaopingC, HZ, ZL, XiangC, and WZ designed the study and revised the manuscript. All the authors contributed to the final paper.

### Conflict of interest statement

The authors declare that the research was conducted in the absence of any commercial or financial relationships that could be construed as a potential conflict of interest. The reviewer TS and handling editor declared their shared affiliation.
