# Supplementary material for: PSORS1C1 Hypomethylation Is Associated with Allopurinol-Induced Severe Cutaneous Adverse Reactions during Disease Onset Period: A Multicenter Retrospective Case-Control Clinical Study in Han Chinese
Source: Front Pharmacol. 2018 Jan 17;8:923. doi: 10.3389/fphar.2017.00923 (PMC5776094; doi:10.3389/fphar.2017.00923)
Supplement: Supplementary file 1 [file Table1.doc]

**Supplementary FIGURE 1. Flowchart showing the design and analysis methods of the study.**

**Supplementary FIGURE 2. Pyrosequencing validation of differentially methylated CpG (cytosine-guanine dinucleotide) loci** ***PSORS1C1* cg24926791 and *DUSP22* cg03395511.** (A) *PSORS1C1* cg24926791 and *DUSP22* cg03395511 CpG sites were amplified by PCR after bisulfite conversion of genomic DNA. (B) Pyrosequencing chtomatogram of *PSORS1C1* cg24926791 CpG site in an independent cohorts. Three methylated sites are highlighted in grey, one quality control site is highlighted in yellow. The quality control site should not show any waves in complete bisulfite conversion. In grey area, position 1 is the target detecting site (array site), while the next sites are two areas after it (CpG1 and CpG2). The sequence result is presented in the “sequence to analyze” area. Y denotes the methylated site. In the chtomatogram, some bases of T, A are added as quality control. (C) Pyrosequencing chromatogram of *DUSP22* cg03395511 CpG site in an independent cohorts. One quality control site is highlighted in yellow, and two methylated sites are highlighted in grey, with the first one as the target detecting site.

**Supplementary Table 1. PCR and sequencing primers for two CpG sites by using bisulfate-converted genomic DNA**

| **Probe** | **Gene** | **PCR forward primer** | **PCR reverse primer** | **Sequencing primer** |
| --- | --- | --- | --- | --- |
| cg24926791 | *PSORS1C1* | GTGGGGTTTTTATAGGGATAAGT | CTTCACACTCCCTACTACTAA | ATTTTGTTTTTTTTGGGATG |
| cg03395511 | *DUSP22* | GGTAGGGGGTTTTTAGATTTTTT | CCCCCAACCTAAATCTACC | CCCAAAAACCAAACCTCT |

**Supplementary Table 2. Sample characteristics of 40 allopurinol-SCARs and 48 allopurinol-tolerants used as validation cohorts**

| **Characteristics** | **DRESS (n=11)** | **TEN (n=4)** | **SJS/TEN overlap (n=4)** | **SJS (n=21)** | **SCARs (n=40) ^ʃ^** | **Tolerant controls (n=48) ^¢^** |
| --- | --- | --- | --- | --- | --- | --- |
| **AGE (YEAR)** |  |  |  |  |  |  |
| Mean ± SD; range | 60.44±12.38; 39-79 | 63±13.37; 49-81 | 63.2±17.71; 33-80 | 52.6±16.48; 23-84 | 57±15.04; 23-84 | 54.62±14.50; 20-80 |
| **GENDER** |  |  |  |  |  |  |
| Male, n (%) | 10 (90.91) | 2 (50) | 3 (75) | 16 (76.19) | 31 (77.5) | 45 (93.75) |
| Female, n (%) | 1 (9.09) | 2 (50) | 1 (25) | 5 (23.81) | 9 (22.5) | 3 (6.25) |
| **ALLOPURINOL EXPOSURE DOSE (mg/DAY)** |  |  |  |  |  |  |
| Mean ± SD; range | 100 | 100 | 100 | 118±62.92; 50-300 | 110±47.02; 50-300 | 181±91.06; 100-300 |
| **ALLOPURINOL EXPOSURE TIME (DAYS)** |  |  |  |  |  |  |
| Mean ± SD; range | 20.08±9.85; 1-38 | 25.75±9.71; 15-37 | 15.67±3.51; 12-19 | 24.48±14.82; 8-64 | 22.56±12.46; 1-64 | 939.65±1615.29; 60-6570 |

“**^ʃ^**”: 36 *HLA-B*5801* positive and 4 *HLA-B*5801* negative subjects, “**^¢^**”: 6 *HLA-B*5801* positive and *42 HLA-B*5801* negative subjects, all *HLA-B*5801* positive individuals carried one copy of the allele.

**Supplementary Table 3. Pyrosequencing validation of methylation levels for *DUSP22*** **cg03395511 CpG site in an independent 40 allopurinol-SCARs and 48 allopurinol-tolerants cohort**

| Allopurinol-SCARs | Array site | Allopurinol-tolerants | Array site |
| --- | --- | --- | --- |
| DRESS | 0.37 | AT | 0.54 |
| DRESS | 0.39 | AT | 0.34 |
| DRESS | 0.4 | AT | 0.5 |
| DRESS | 0.4 | AT | 0.36 |
| DRESS | 0.47 | AT | 0.38 |
| DRESS | 0.4 | AT | 0.28 |
| DRESS | 0.41 | AT | 0.4 |
| DRESS | 0.46 | AT | 0.37 |
| DRESS | 0.32 | AT | 0.39 |
| DRESS | 0.39 | AT | 0.4 |
| DRESS | 0.35 | AT | 0.52 |
| SJS | 0.33 | AT | 0.38 |
| SJS | 0.32 | AT | 0.39 |
| SJS | 0.3 | AT | 0.3 |
| SJS | 0.3 | AT | 0.38 |
| SJS | 0.32 | AT | 0.36 |
| SJS | 0.39 | AT | 0.45 |
| SJS | 0.45 | AT | 0.39 |
| SJS | 0.4 | AT | 0.52 |
| SJS | 0.31 | AT | 0.35 |
| SJS | 0.41 | AT | 0.35 |
| SJS | 0.48 | AT | 0.37 |
| SJS | 0.42 | AT | 0.38 |
| SJS | 0.34 | AT | 0.55 |
| SJS | 0.35 | AT | 0.32 |
| SJS | 0.29 | AT | 0.46 |
| SJS | 0.39 | AT | 0.38 |
| SJS | 0.42 | AT | 0.53 |
| SJS | 0.44 | AT | 0.49 |
| SJS | 0.41 | AT | 0.3 |
| SJS | 0.38 | AT | 0.35 |
| SJS | 0.36 | AT | 0.39 |
| SJS/TEN overlap | 0.43 | AT | 0.38 |
| SJS/TEN overlap | 0.42 | AT | 0.38 |
| SJS/TEN overlap | 0.54 | AT | 0.41 |
| SJS/TEN overlap | 0.48 | AT | 0.37 |
| TEN | 0.36 | AT | 0.36 |
| TEN | 0.35 | AT | 0.37 |
| TEN | 0.35 | AT | 0.53 |
| TEN | 0.32 | AT | 0.37 |
|  |  | AT | - |
|  |  | AT | - |
|  |  | AT | - |
|  |  | AT | - |
|  |  | AT | - |
|  |  | AT | - |
|  |  | AT | - |
|  |  | AT | - |

AT, allopurinol-tolerants; “-”, missing data (did not pass quality control).
