# Supplementary figures and images for: PSORS1C1 Hypomethylation Is Associated with Allopurinol-Induced Severe Cutaneous Adverse Reactions during Disease Onset Period: A Multicenter Retrospective Case-Control Clinical Study in Han Chinese
Source: Front Pharmacol. 2018 Jan 17;8:923. doi: 10.3389/fphar.2017.00923 (PMC5776094; doi:10.3389/fphar.2017.00923)

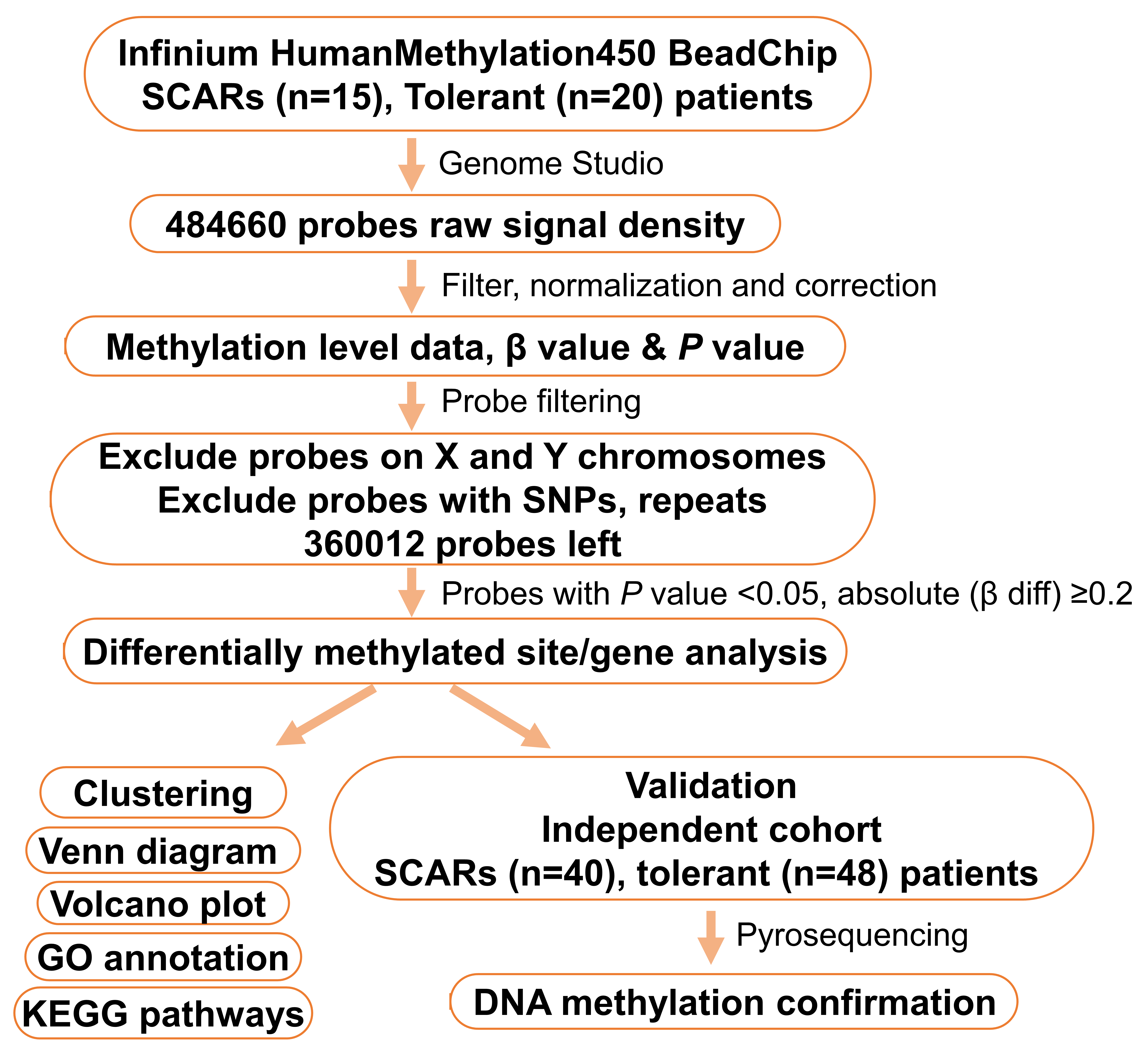

Supplement: Supplementary file 2 [file Image1.tif]

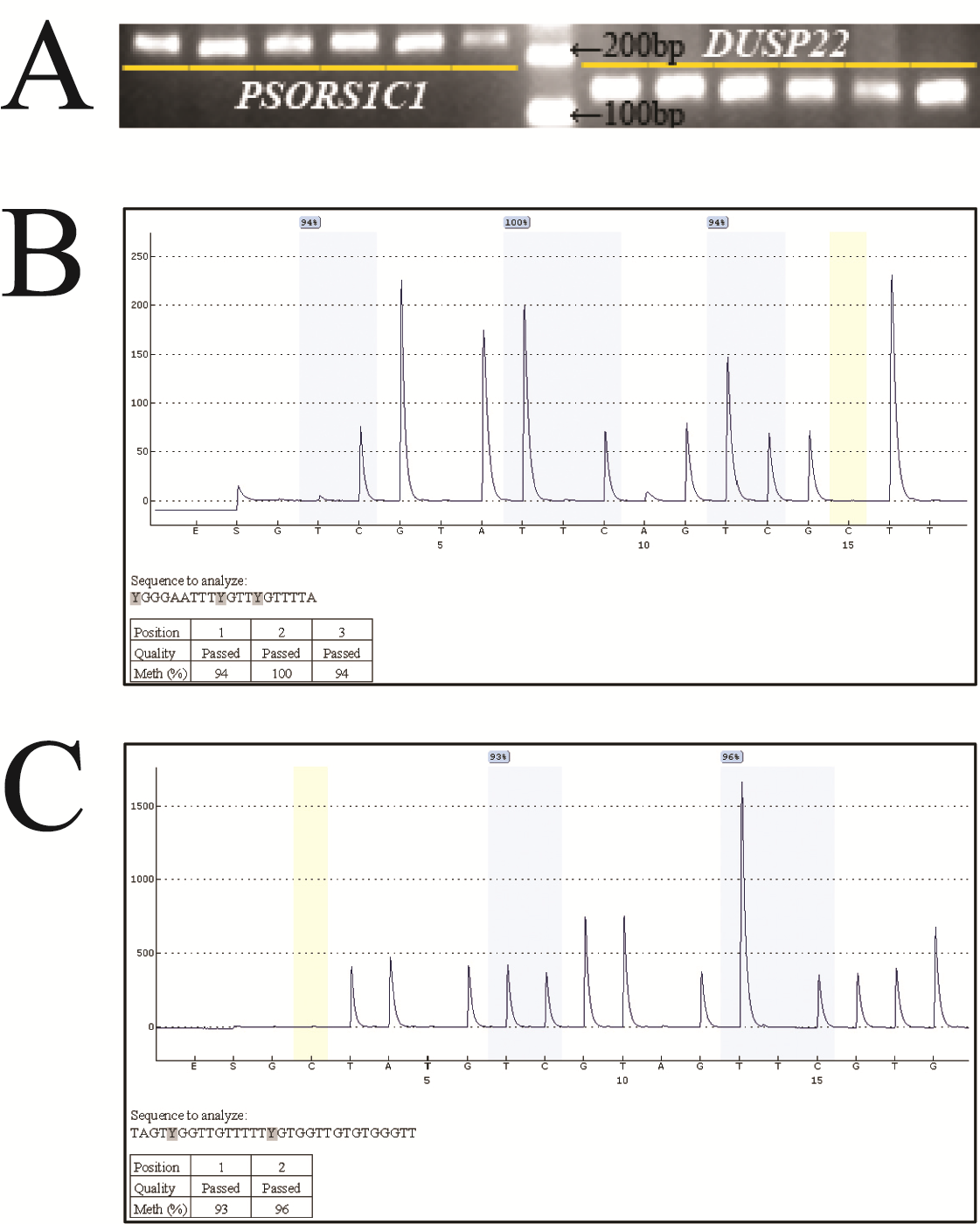

Supplement: Supplementary file 3 [file Image2.tif]
